# Kalium: a database of potassium channel toxins from scorpion venom

**DOI:** 10.1093/database/baw056

**Published:** 2016-04-16

**Authors:** Alexey I. Kuzmenkov, Nikolay A. Krylov, Anton O. Chugunov, Eugene V. Grishin, Alexander A. Vassilevski

**Affiliations:** ^1^Shemyakin-Ovchinnikov Institute of Bioorganic Chemistry, Russian Academy of Sciences, Moscow 117997, Russia; ^2^Joint Supercomputer Center, Russian Academy of Sciences, Moscow 119991, Russia

## Abstract

Kalium (http://kaliumdb.org/) is a manually curated database that accumulates data on potassium channel toxins purified from scorpion venom (KTx). This database is an open-access resource, and provides easy access to pages of other databases of interest, such as UniProt, PDB, NCBI Taxonomy Browser, and PubMed. General achievements of Kalium are a strict and easy regulation of KTx classification based on the unified nomenclature supported by researchers in the field, removal of peptides with partial sequence and entries supported by transcriptomic information only, classification of β-family toxins, and addition of a novel λ-family. Molecules presented in the database can be processed by the Clustal Omega server using a one-click option. Molecular masses of mature peptides are calculated and available activity data are compiled for all KTx. We believe that Kalium is not only of high interest to professional toxinologists, but also of general utility to the scientific community.

**Database URL**: http://kaliumdb.org/

## Introduction

Ion channels are an indispensable feature of life on Earth (1). Playing leading roles in hormone secretion, cell division and motility, muscle contraction, sense perception and brain functioning, these proteins are one of the primary targets for drug development (2). Exploration of ion channel structure and function is one of the important challenges to biochemistry and physiology, and researchers apply a variety of pharmacological agents as molecular tools to aid their studies.

Among ion channels, potassium channels form the most populated and diversified superfamily. These channels are found in all living organisms from bacteria to humans (3). Their fundamental function in the human body is to set the resting potential and shape the action potential in nerves and muscles (1). Most potassium channels are tetramers of principal α-subunits (heteromeric assemblies are more common) (4), which define their major properties and are often supplemented by auxiliary β-subunits (5). There are 78 genes encoding α-subunits of potassium channels in the human genome allocated to five groups: K_ir_, K_2P_, K_V_ and two groups of K_Ca_, as recommended by the International Union of Basic and Clinical Pharmacology (IUPHAR) (6–9).

Research into potassium channels relies in a large part on the availability of precise molecular tools that can be used to modulate their activity in a desired way. The diversity of potassium channel ligands may be divided into two large groups, i.e. pore blockers that physically occlude the channel pore, and gating modifiers that affect channel properties otherwise (10). From a chemical standpoint, among potassium channel ligands we note three major classes: metal ions, low-molecular-mass substances and polypeptides (11).

Perhaps the most diversified source of potassium channel ligands is scorpion venom: out of ∼400 polypeptide ligands found in UniProt, 250 are scorpion toxins (12). All known scorpion toxins affecting potassium channels (KTx) are peptides that act as channel pore blockers (13). KTx are utilized to localize the channels in biological samples, isolate these proteins and investigate their pharmacology. There is increasing enthusiasm in the development of drugs from KTx, since more potassium channels become validated as drug targets (14, 15).

KTx are built of ∼20–75 amino acid residues and contain 2–4 disulfide bridges. There are five structural folds found in KTx. (i) An overwhelming majority of KTx conform to the cysteine-stabilized α-helix-β-sheet fold (CSα/β), also typical of sodium channel toxins and chlorotoxin-like peptides from scorpion venom (12, 16, 17). (ii) κ-Hefutoxin and related peptides contain two parallel α- helices connected with two disulfide bonds, and the corresponding fold is designated CSα/α 2(C-C) (18). (iii) An alternative pattern of disulfide bond formation is noted in some recently discovered KTx that are homologous to CSα/β toxins but assume the cysteine-stabilized helix-loop-helix fold named CSα/α 3(C-C) (19). Moreover, KTx are known with (iv) the Kunitz-type fold characteristic of serine protease inhibitors (20), and (v) the inhibitor cystine knot (ICK) fold common to spider toxins (21).

In 1999, leading scientists in the field proposed a so-called unified nomenclature to address and systematize the growing number of known KTx (22). With certain modification, this nomenclature lives to date and is supported by the community (23). Today all KTx are proposed to be grouped into 6 families based on homology, 3D folding pattern, and activity. CSα/β toxins are divided into three families: α-KTx (∼20–40 residues), β-KTx (∼45–75 residues) and γ-KTx (affecting a particular subset of K_V_, so-called ERG channels). CSα/α toxins are placed in the κ-KTx family, Kunitz toxins are named δ-KTx, and most recently ICK toxins have been proposed to constitute the λ-KTx family. Each family comprises subfamilies that in turn consist of individual KTx grouped by homology (24, 25). The nomenclature uses two numbers to identify each toxin, one to specify the subfamily, and the other to specify an individual member. For example, the seminal charybdotoxin is named as α-KTx 1.1 (family α-KTx, subfamily 1, the first member). As new KTx are discovered, subfamily and individual toxin numbers are afforded in a chronological order.

KTx are often active at nanomolar or even subnanomolar concentrations. Some display marked selectivity with respect to particular subtypes of K_V_ and K_Ca_, but none were reported to be active against K_ir_ or K_2P_ (26). Some α-KTx bind to the pore of K_V_ or K_Ca_ via amino acid residues located in their β-hairpin (27, 28). Some other KTx including γ-KTx interact with the channel vestibule via amino acid residues of their α-helix (29). Many KTx contain the so-called ‘functional dyad’ composed of two highly conserved and functionally important amino acid residues, the first being lysine—its side chain enters the pore as visualized in the crystal structure of charybdotoxin with a K_V_ (30), and the second being tyrosine, phenylalanine, or leucine (31). Alternatively, KTx may contain a ‘ring of basic residues’ that forms salt bridges with the negatively charged residues of the channel (32, 33). Not only the residues of the dyad or the ring determine the mode of toxin binding to the channel. Rather, ‘complementary’ surfaces of toxins and channels are involved in close contact during complex formation (34).

There is growing need for a curated database on KTx. Now that there are so many KTx, systematization is an obvious demand. We believe that a modified unified nomenclature of KTx is a robust tool for toxin systematics. But there should also exist an easily accessible up-to-date list of afforded names. Lack of such has already caused a lot of misunderstanding. Moreover, a repository offering all available structural and functional data on KTx and enabling their easy comparison and data retrieval is an important factor for further progress. At present none of these requirements are met by the existing public databases. There is also considerable concern regarding the current state of KTx systematization as presented in UniProt (35).

Here we present Kalium, a manually curated database, which offers a comprehensive list of all known KTx and compiles all available structural and activity data on these substances. We also take this chance to refine the KTx systematics and bring it up to date with the help of the leading experts. Further database development is streamlined by automated and manual cross-interactions with UniProt.

## Data sources and curation

The compiled data on all publically available sequences of potassium channel blockers from scorpion venom were obtained from UniProtKB. Available PDB structures with links to the RCSB Protein Data Bank and location of disulfide bonds were also extracted from UniProt. The data set was then manually filtered and refined, including the following steps: removal of peptides with partial sequence, removal of entries supported by transcriptomic information only, classification of toxins belonging to the β-family, and addition of a novel λ-family.

Partially sequenced peptides were excluded because they cannot be used straightforwardly for nomenclature or in further research and bring confusion to the entire classification. Sequences obtained from scorpion transcriptomes without verification on protein level were also left out because (i) they are of less interest for researchers, (ii) there is differential presence or absence of transcriptomic entries from different scorpions in UniProt-supported toxin classification and (iii) fast growing numbers of transcriptomic studies and low accuracy of such data.

Toxins comprising the β-family were classified similarly to molecules from the α-family: β-KTx 1.1, β-KTx 1.2 etc. A new λ-family containing toxins with the ICK motif was introduced into the overall classification with the same nomenclature structure.

For every curated entry molecular mass calculation was performed taking into consideration the co- and posttranslational modifications (cleavage of signal and propeptides, N-terminal cyclization of glutamine, C-terminal amidation and disulfide bridge formation). Tables of amino acid masses and modifications from the Expasy server were used for calculations: http://web.expasy.org/findmod/findmod_masses.html#aas—amino acid molecular masses, http://web.expasy.org/findmod/AMID.html—amidation of C-terminal amino acids, http://web.expasy.org/findmod/PYRRE.html—cyclization of N-terminal glutamine into pyroglutamate. Disulfide bonds were taken into account by subtracting two proton masses from the mass of two cysteines.

**Figure 1.**
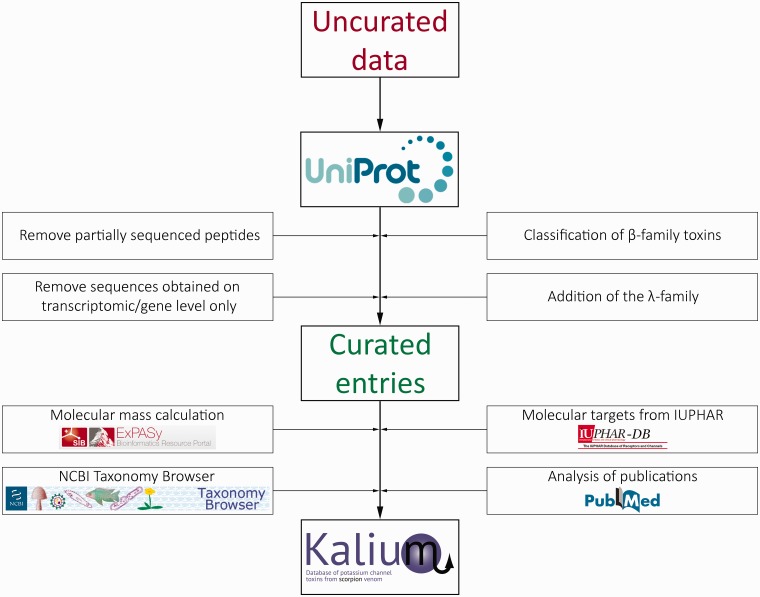
Schematic representation of the data stream and curation process in Kalium.

Further, every scorpion Latin name was linked to a valid species entry on the NCBI Taxonomy Browser. Comprehensive activity data were added manually from literature and linked to corresponding references in PubMed. Molecular target nomenclature was adopted as recommended by IUPHAR. The data stream and curation process in Kalium are presented in Figure 1.

## Architecture and implementation

Kalium is an OpenUI 5 Model-View-Controller web application that uses a Django server-side framework and SQLite3 database. The web interface utilizes Asynchronous JavaScript and XML, and dynamically generated HTML5 pages. Database curation is based on a standard Django web admin interface. The main interface element of Kalium is the table with data on toxins, initially sorted according to ‘Name’. The table supports searching, multi-column ordering and filtering, multi-row selection and sequence alignment on the Clustal Omega web server. All major browsers are supported.

## Kalium structure and features

**Figure 2.**
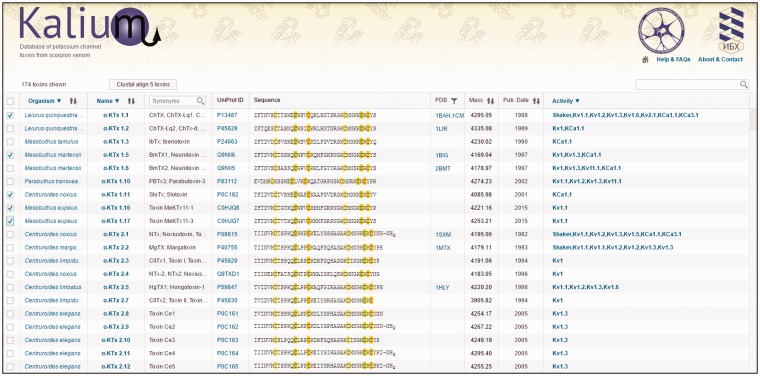
Initial window of Kalium. Top panel consists of the database logo (implementing the Home-button function), and links to ‘About & Contact’ and ‘Help & FAQs’ pages. The second panel contains an indicator of the selected entries number, the ‘Clustal’ button, and a search bar. The main body of the database is presented by a table consisting of 10 columns (as described in ‘Kalium structure and features’).

The main window of Kalium is presented by one large table, in which all data about potassium channel toxins from scorpions are assembled. ‘About & Contact’ and ‘Help & FAQs’ located at the top right corner link to pages that contain information about developers and tips. Control elements of the table are located in headers and function to filter information of interest as discussed below (Figure 2).

- **Selection**—permits to select one or more entries and send them to the Clustal Omega server. For all entries selection, users need to click once on the column header. After selection, click the ‘Clustal’ button to show results.
- **Organism**—scorpion species name according to current biological classification. One click on the column header opens a menu where users can choose one or more scorpion species to filter the full data set. Scorpion Latin names are linked to the NCBI Taxonomy Browser ensuring a valid classification.
- **Name**—toxins are shown grouped in families and subfamilies according to an updated Tytgat-Possani nomenclature. The general improvements of current classification are removal of entries with partial amino acid sequences and sequences established on transcriptomic level only. Select family from the drop-down menu. Toxin card (see below) opens when clicked on toxin name.
- **Synonyms:** trivial names of toxins. Many scientists identify certain molecules using trivial names only, therefore their presence is a necessity. Search option is available at the column header.
- **UniProt ID:** UniProt ID is shown, click to switch to corresponding UniProt page.
- **Sequence:** polypeptide sequence of mature toxin presented in one-letter code. Cysteine residues are marked; different colors indicate the disulfide bond connectivity.
- **PDB:** Shows PDB ID if available, click to switch to corresponding PDB page. Use the filter button to show entries with resolved spatial structure only.
- **Mass:** molecular masses of mature toxins are calculated taking post-translational modifications into account.
- **Publication date:** the date when the molecule was first published.
- **Activity data:** information about activities on different potassium channels. All tested targets are shown in the list. Toxin card can be opened for detailed information. Use the header to filter toxins according to specific targets.

## Toxin card

**Figure 3.**
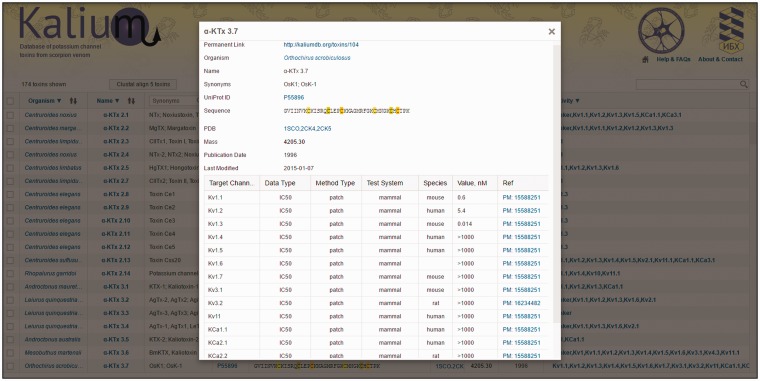
Toxin card overview. α-KTx 3.7 (OSK1) is taken as an example. All information present in the general table is duplicated here with certain additions (as described in ‘Toxin card’). Activity data are summarized in a table located to the bottom of the card.

For every toxin, detailed information is summarized in the toxin card. Here all information presented in the general table is duplicated with addition of some points as explained below (Figure 3).

- **Permanent link:** can be used for citation.
- **Raw sequence:** presents precursor sequence if available.
- **Last modified**: date of the latest modification to the entry.

The activity data are accumulated in a table containing the following columns:

- **Target channel**: potassium channels that were used for toxin activity measurements.
- **Data type**: specifies the type of data reported: dissociation constant (K_d_), inhibition constant (Ki), half-maximal inhibitory concentration (IC_50_), or half-maximal effective concentration (EC_50_).
- **Method type**: specifies the experimental method applied: radio, radioligand-binding assay; flux, rubidium/thallium efflux assay; patch, electrophysiology using patch-clamp technique; volt, electrophysiology using voltage-clamp technique.
- **Test system**: specifies the cell type used for channel expression: insect, *Xenopus* oocyte, or mammalian.
- **Species**: specifies origin of ion channel that was used for measurements. The most common channels belong to fly, rat, mouse, and human organisms. Blank means that the origin of ion channel was not specified in the publication.
- **Value, nM**: numeric value of peptide activity (K_d_, Ki, IC_50_ or EC_50_) presented in nM. These data are collected manually from literature. Values are shown in the following formats:
  - *X* – K_d_, Ki, IC_50_, or EC_50_ value in nM;
  - >*X* – toxin had no effect at up to *X* value
  - *X*/*Y* – means that toxin at concentration *X* reduced ion current through the channels by *Y* percent.
- **Reference:** reference article with PubMed ID.

## Kalium is accessible in the web interface

Kalium is a freely accessible public database placed in the .org domain (http://kaliumdb.org/). The database is updated automatically from UniProtKB. Every update seeks manual curation; administrator is asked to confirm the newly uploaded information and add activity data.

## Current status of Kalium and future directions

Kalium includes all potassium channel blockers purified from scorpion venom to date. The data set includes molecules for which information on protein level is available, and currently 174 in total. We plan to expand the database by adding potassium channel ligands from other sources such as snakes, cone snails, bees, sea anemones and spiders.
